# Non-alcoholic fatty liver disease and cardiovascular disease: assessing the evidence for causality

**DOI:** 10.1007/s00125-019-05024-3

**Published:** 2019-11-11

**Authors:** Martijn C. G. J. Brouwers, Nynke Simons, Coen D. A. Stehouwer, Aaron Isaacs

**Affiliations:** 1 Division of Endocrinology and Metabolic Disease, Department of Internal Medicine, Maastricht University Medical Centre, P. Debyelaan 25, 6229 HX Maastricht, the Netherlands; 2 Cardiovascular Research Institute Maastricht (CARIM), Maastricht University, Maastricht, the Netherlands; 3 Laboratory for Metabolism and Vascular Medicine, Division of General Internal Medicine, Department of Internal Medicine, Maastricht University Medical Centre, Maastricht, the Netherlands; 4 Division of General Internal Medicine, Department of Internal Medicine, Maastricht University Medical Centre, Maastricht, the Netherlands; 5 Maastricht Centre for Systems Biology (MaCSBio), Maastricht University, Maastricht, the Netherlands; 6 Department of Biochemistry, Maastricht University, Maastricht, the Netherlands

**Keywords:** Cardiovascular disease, Coronary artery disease, GCKR, Mendelian randomisation, Non-alcoholic fatty liver disease, Non-alcoholic steatohepatitis, *PNPLA3*, Review, *TM6SF2*

## Abstract

Non-alcoholic fatty liver disease (NAFLD) is highly prevalent among individuals with type 2 diabetes. Although epidemiological studies have shown that NAFLD is associated with cardiovascular disease (CVD), it remains unknown whether NAFLD is an active contributor or an innocent bystander. Plasma lipids, low-grade inflammation, impaired fibrinolysis and hepatokines are potential mediators of the relationship between NAFLD and CVD. The Mendelian randomisation approach can help to make causal inferences. Studies that used common variants in *PNPLA3*, *TM6SF2* and *GCKR* as instruments to investigate the relationship between NAFLD and coronary artery disease (CAD) have reported contrasting results. Variants in *PNPLA3* and *TM6SF2* were found to protect against CAD, whereas variants in *GCKR* were positively associated with CAD. Since all three genes have been associated with non-alcoholic steatohepatitis, the second stage of NAFLD, the question of whether low-grade inflammation is an important mediator of the relationship between NAFLD and CAD arises. In contrast, the differential effects of these genes on plasma lipids (i.e. lipid-lowering for *PNPLA3* and *TM6SF2*, and lipid-raising for *GCKR*) strongly suggest that plasma lipids account for their differential effects on CAD risk. This concept has recently been confirmed in an extended set of 12 NAFLD susceptibility genes. From these studies it appears that plasma lipids are an important mediator between NAFLD and CVD risk. These findings have important clinical implications, particularly for the design of anti-NAFLD drugs that also affect lipid metabolism.

**Electronic supplementary material:**

The online version of this article (10.1007/s00125-019-05024-3) contains a slideset of the figures for download, which is available to authorised users.

## Introduction

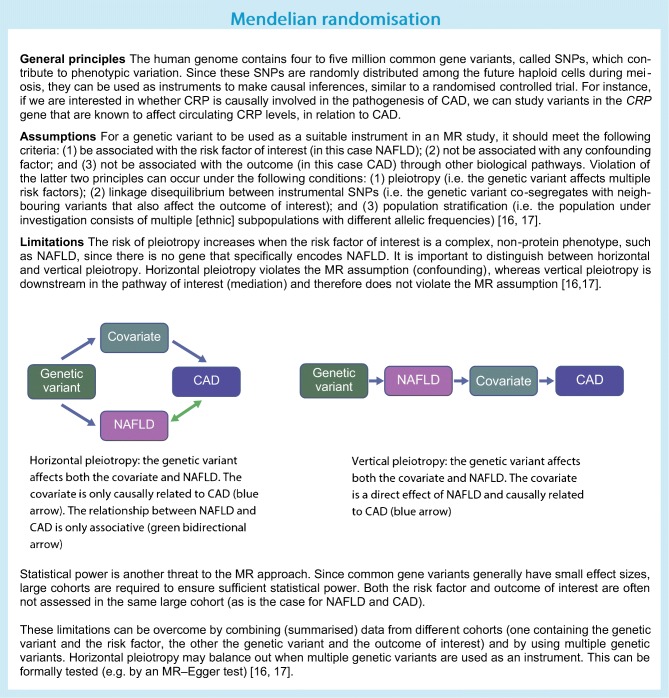



Non-alcoholic fatty liver disease (NAFLD) is a frequently encountered phenomenon in type 2 diabetes. It is a histological spectrum consisting of hepatic fat accumulation (‘simple steatosis’), non-alcoholic steatohepatitis (NASH), fibrosis and cirrhosis, occurring in the absence of excessive alcohol intake. It has been estimated that the global prevalence of NAFLD is 25% and that 23% of affected individuals have type 2 diabetes [1].

The pathogenesis of NAFLD involves a complex interaction between genetic and environmental factors. The accumulation of hepatic fat is the consequence of an imbalance between the influx of fat (i.e. fatty acids from adipose tissue or diet and de novo lipogenesis from glucose) and the efflux of fat (i.e. β-oxidation and synthesis of VLDL). Stable isotope studies have demonstrated that an increased fatty acid flux and de novo lipogenesis are the two principal processes contributing to hepatic fat accumulation in individuals with NAFLD [2]. Although hepatic fat accumulation is a prerequisite for the development of NASH, not all individuals with simple steatosis proceed to this stage. Lipotoxicity, the key driver behind the development of NASH, is determined by the following factors: (1) both the quantity and type of lipids that accumulate; and (2) the ability of the liver to defend against lipotoxicity [3].

Although NAFLD is a risk factor for end-stage liver disease and hepatocellular carcinoma (it is projected to be the principal cause of liver transplantation by 2025 [4]), individuals with NAFLD mostly die from cardiovascular disease (CVD) [5]. A previous meta-analysis demonstrated that the risk of developing a fatal and/or non-fatal CVD event is 64% higher in individuals with vs without NAFLD [6]. Of note, in the majority of the studies that were included in this meta-analysis, the diagnosis of NAFLD was established by either ultrasound or computed tomography [6], which are only capable of diagnosing simple steatosis, not advanced stages of NAFLD. In contrast, one of the few studies that included histologically confirmed NAFLD, and showed that advanced liver fibrosis specifically accounted for the greater CVD risk, was limited by a relatively small sample size and a selected population that underwent a liver biopsy [7].

**Fig. 1:**
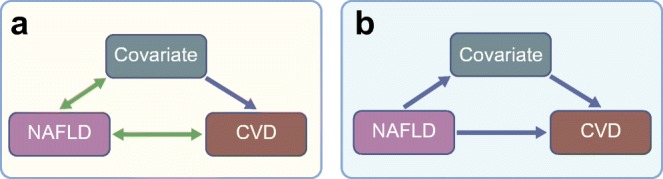
Confounding and mediation. The relationship between NAFLD and CVD, as reported in observational studies, can be explained by confounding or mediation. (**a**) In confounding, there is a covariate that is (non-causally) associated with NAFLD (green bidirectional arrow) and causally related to CVD (blue arrow), which explains the non-causal association between NAFLD and CVD. In other words, NAFLD is an innocent bystander. (**b**) In mediation, there is a covariate that is the direct result of NAFLD and causally relates to CVD. In other words, NAFLD is an active contributor to CVD risk, mediated by the covariate. This figure is available as part of a downloadable slideset

There is an ongoing discussion on whether NAFLD is truly an active contributor or an innocent bystander in the development of CVD, as recently reviewed [8]. It is often presumed that the association between NAFLD and CVD is confounded by NAFLD-related factors (i.e. the association of NAFLD with other factors related to the metabolic syndrome, such as dyslipidaemia, hypertension and type 2 diabetes) and this could theoretically explain the association between NAFLD and CVD [8–10]. Of interest, NAFLD was found to be the strongest determinant of increased intima–media thickness, independent of the potential confounding effects of age, sex, visceral fat mass, state of hyperglycaemia, insulin resistance and insulin secretion, in individuals with impaired fasting glucose and/or impaired glucose tolerance [11]. It should, however, be noted that some of these factors are not necessarily confounders but instead could act as mediators of the relationship between NAFLD and CVD (Fig. 1). Since NAFLD and CVD are biologically distant traits, they must be linked through mediating factors. We will argue that plasma lipids act as an important mediator between NAFLD and CVD.

Therefore, the present review has several aims: (1) to elaborate on potential mediators of the relationship between NAFLD and CVD; (2) to provide experimental evidence for a causal relationship between NAFLD and CVD; and (3) to discuss clinical implications.

## Potential mediators of the association between NAFLD and CVD

**Fig. 2:**
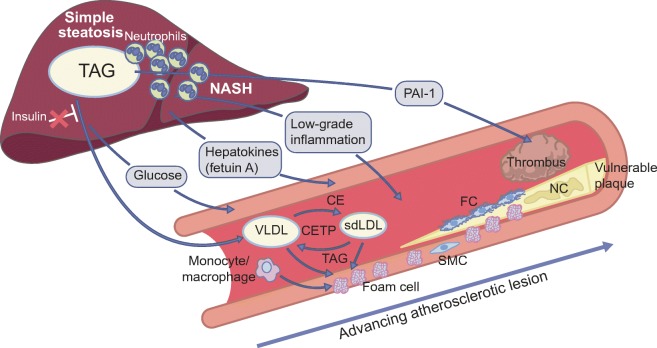
Biologically plausible mechanisms that link NAFLD to the pathogenesis of an atherosclerotic lesion. The combination of endothelial dysfunction and lipoprotein retention in the arterial wall triggers a low-grade inflammatory response, which results in the accumulation of lipid-loaded, monocyte-derived macrophages (‘foam cells’) and the proliferation of smooth muscle cells (SMCs). This newly formed lesion is covered by a thin fibrous cap (FC). The so-called ‘vulnerable plaque’ consists of a necrotic core (NC) and a fibrous cap, which makes it prone to rupture. The subsequent exposure of pro-thrombotic, necrotic material to the blood stream results in acute thrombosis and occlusion of the artery. Simple steatosis (shown on the left side of the liver) and insulin resistance drive overproduction of triacylglycerol-rich VLDL particles. Small-dense LDL (sdLDL) particles, the most atherogenic lipoproteins, are formed by the CETP-mediated exchange of cholesteryl ester (CE) and triacylglycerols (TAG) between VLDL and LDL particles. NASH (shown on the right side of the liver) could theoretically contribute to the low-grade inflammatory environment that is present in the atherosclerotic lesion. Finally, NAFLD (potentially steatosis, NASH or both) contributes to higher circulating PAI-1 levels, which impede the resolution of a thrombus. Other factors that may affect atherogenesis include insulin resistance-induced overproduction of glucose (which promotes monocyte/macrophage adhesion, SMC chemokine secretion and expression of an inflammatory phenotype in macrophages) and fetuin A secretion (which induces low-grade inflammation). This figure is available as part of a downloadable slideset

The pathogenesis of an atherosclerotic plaque, the pathological lesion that is responsible for a cardiovascular event, consists of several stages (Fig. 2), as reviewed in detail elsewhere [12]. NAFLD could theoretically contribute to all of these stages.

### NAFLD and dyslipidaemia

Individuals with NAFLD have a typical plasma lipid pattern characterised by elevated plasma triacylglycerols, low HDL-cholesterol and a high number of circulating small-dense LDL particles [13]. Stable isotope studies have shown that NAFLD is associated with (insulin-resistant) overproduction of triacylglycerol-rich VLDL particles [14]. In plasma, triacylglycerols from VLDL particles are exchanged for cholesteryl esters from LDL and HDL particles, a process mediated by cholesteryl ester transfer protein (CETP). Once these triacylglycerols have been hydrolysed by hepatic lipase, both LDL and HDL particles become small and cholesterol-depleted [15].

The Mendelian randomisation (MR) approach can help to make causal inferences, as summarised in Text box 1 and comprehensively described elsewhere [16, 17]. Previous MR studies have demonstrated that plasma triacylglycerols are causal in the pathogenesis of coronary artery disease (CAD) [18], whereas low plasma HDL-cholesterol levels do not necessarily play an active role in the pathogenesis of CVD [19]. Experimental studies have shown that small-dense LDL particles are highly atherogenic [20].

### NAFLD and low-grade inflammation

NASH could theoretically create a systemic, low-grade inflammatory environment that promotes the pathogenesis of atherosclerosis by secreting cytokines and acute-phase proteins [8, 9]. Although C-reactive protein (CRP) is a liver-specific protein that has been associated with both NASH and CVD [21, 22], MR studies have revealed that CRP is merely a biomarker, not a mediator, of CVD risk [22].

The clinical relevance of low-grade inflammation in the pathogenesis of CVD has recently been demonstrated unequivocally by the Canakinumab Anti-inflammatory Thrombosis Outcome Study (CANTOS), in which treatment with canakinumab, a monoclonal antibody targeting IL-1β, reduced recurrent CVD events independent of lipid lowering [23]. Of interest, IL-1β also plays an active role in the pathogenesis of NASH in different animal models [24]. It is not known, however, whether IL-1β mediates the association between NASH and CVD.

### NAFLD and thrombosis

The rupture of a vulnerable atherosclerotic lesion and subsequent thrombosis is clinically manifested as an acute ischaemic event. Plasminogen activator inhibitor type 1 (PAI-1) is an important inhibitor of the fibrinolytic system and, hence, the resolution of a thrombus. Previous studies have shown that PAI-1 levels are elevated in individuals with NAFLD and that the liver is a principal determinant of plasma PAI-1 levels [25]. MR studies have demonstrated that elevated PAI-1 levels have a causal effect on CAD [26].

### Other mediators

NAFLD has been associated with insulin-resistant, endogenous glucose production and, consequently, incident type 2 diabetes [27, 28]. MR studies have shown that plasma glucose is causally related to CAD [29], possibly by promoting monocyte/macrophage adhesion to the endothelium, chemokine secretion by vascular smooth muscle cells, and expression of an inflammatory phenotype in macrophages [30]. More recently, the so-called hepatokines have emerged as potential mediators of cardiometabolic complications in NAFLD [31]. Of these, fetuin A has been associated with CVD [32]. Causality was inferred by one MR study [33] but this was not replicated in an MR analysis that included prospective studies [34]. Experimental studies have shown that fetuin A induces low-grade inflammation in concert with fatty acids [35].

## Assessing the evidence for a causal relationship between NAFLD and CVD

### Intervention studies

Currently, there are two drugs that have been shown to affect both NAFLD and CVD (i.e. pioglitazone and liraglutide). Pioglitazone is highly effective in the treatment of biopsy-proven NASH (number needed to treat to resolve NASH after 18 months’ treatment: ~3) [36]. The Prospective Pioglitazone Clinical Trial in Macrovascular events (PROactive) study showed a benefit for pioglitazone with respect to the secondary outcome (a composite of all-cause mortality, non-fatal myocardial infarction and stroke) [37]. More recently, pioglitazone treatment in insulin-resistant patients with recent ischaemic stroke or transient ischaemic attack (but without type 2 diabetes) resulted in a significant reduction in the occurrence of cardiovascular events [38].

Liraglutide has a similar effect on NASH (number needed to treat to resolve NASH after 48 weeks’ treatment: ~3) [39] and the Liraglutide Effect and Action in Diabetes: Evaluation of Cardiovascular Outcome Results (LEADER) trial showed that liraglutide was superior to standard care in reducing major adverse cardiovascular events in type 2 diabetes [40].

These interventions, however, do not specifically target NAFLD. It cannot, therefore, be concluded from these studies that NAFLD is causal in explaining CVD risk reduction.

### MR studies

More than 10 years ago, we were the first to show that the heritability of NAFLD, assessed by ultrasound, is 25–35% in dyslipidaemic pedigrees [41]. Subsequent linkage analyses revealed three quantitative trait loci on chromosome 1q42.3, 7p12-21 and 22p13-q11 that were associated with the fatty liver trait [41]. Of note, Romeo and colleagues later identified the gene encoding patatin-like phospholipase domain-containing protein 3 (PNPLA3), located on chromosome 22q13.31, as the first NAFLD susceptibility gene [42], which has been associated with all stages of NAFLD [43].

The common variant in *PNPLA3* (rs738409) was also used as an instrument in the first and at present only MR study to investigate the causal relationship between NAFLD and CAD. That study did not find any association [44]. In fact, we observed that the rs738409 G allele that predisposes to NAFLD conferred a modest protection from CAD in the CARDIoGRAMplusC4D dataset (www.cardiogramplusc4d.org; accessed 23 August 2019), consisting of 60,801 CAD cases and 123,504 controls [45]. This observation was confirmed in the Myocardial Infarction Genetics and CARDIoGRAM Exome Consortia study [46], which only partly overlaps with the CARDIoGRAMplusC4D dataset.

**Fig. 3:**
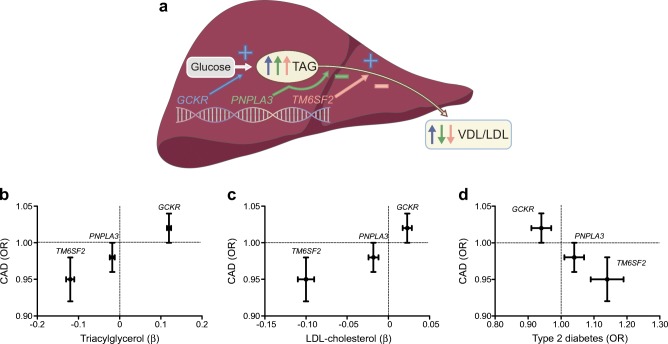
Relationship of *GCKR*, *PNPLA3* and *TM6SF2* with plasma lipids, type 2 diabetes and CAD. (**a**) Variants in *GCKR*, *PNPLA3* and *TM6SF2* contribute to the development of intrahepatic triacylglycerol (TAG) accumulation by greater hepatic glucose uptake and de novo lipogenesis (*GCKR*), impaired lipid-droplet remodelling (*PNPLA3*) and impaired VLDL secretion (*PNPLA3* and *TM6SF2*). As a consequence, they have differential effects on plasma lipid levels. (**b**–**d**) Associations of common variants in *GCKR*, *PNPLA3* and *TM6SF2* with plasma triacylglycerols (**b**), LDL-cholesterol (**c**) and type 2 diabetes (**d**) (on *x*-axes) and CAD (on *y*-axes). Lipid data are derived from [46]; CAD data are derived from [45, 52]; type 2 diabetes data are derived from [50]. Error bars indicate 95% CIs. This figure is available as part of a downloadable slideset

A similar protective effect has been found for the rs58542926 T allele (*TM6SF2*) a more-recently discovered NAFLD susceptibility gene encoding transmembrane 6 superfamily 2 (TM6SF2) [45, 47]. These apparently paradoxical observations may be explained by considering the functions of these two gene products. Although the true function of PNPLA3 remains to be elucidated, it has been suggested that it is involved in lipid droplet remodelling and VLDL production [48]. TM6SF2 is also involved in VLDL production (Fig. 3a) [49]. Indeed, variants in both *PNPLA3* and *TM6SF2* have also been associated with lower plasma lipid levels, both triacylglycerols and LDL-cholesterol [46], which might explain the negative relationship of these SNPs with CAD (Fig. 3b,c). The simultaneous effects of *PNPLA3* and *TM6SF2* on both NAFLD and plasma lipids (through impaired VLDL production) are an example of horizontal pleiotropy. They are, therefore, not perfectly suited as instruments for MR studies, particularly when used in monogenic analyses (Text box 1). Furthermore, more recent studies have shown that the same variants in both *PNPLA3* and *TM6SF2* are also positively associated with type 2 diabetes [46, 50].

The third robust NAFLD susceptibility gene, *GCKR* (encoding liver-specific glucokinase regulatory protein [GKRP]), is involved in de novo lipogenesis (Fig. 3a) [51], one of the principal pathways in the development of NAFLD [2]. In a recent meta-analysis, we showed that common variants in this gene (rs1260326, rs780094 and rs780093, which are all in strong linkage disequilibrium) are modestly associated with CAD (OR per risk allele 1.02 [95% CI 1.00, 1.04]) [52]. Of interest, these genetic variants have also been associated with higher serum triacylglycerols, lower serum HDL-cholesterol and the presence of small-dense LDL particles [51], the lipid phenotype that characterises NAFLD [13]. Since it is believed that this lipid phenotype is a consequence of NAFLD (Fig. 3a) [51], it is an example of vertical pleiotropy (or mediation); the gene effect on lipids is through the liver, which does not invalidate the MR assumptions (Text box 1). It cannot, however, be ruled out that the common variants in *GCKR* also have horizontal pleiotropic effects. Previous studies have shown that these variants also protect against chronic kidney disease and type 2 diabetes [50, 52].

Finally, variants in the membrane-bound *O*-acyltransferase domain-containing 7 gene (*MBOAT7*), which is involved in acyl-chain remodelling of phosphatidylinositols, have consistently been associated with NAFLD [53, 54]. Of interest, the rs641738 T allele was not associated with CAD, nor with plasma lipids or type 2 diabetes [45, 50, 55].

These studies suggest that plasma lipids play an important role in explaining the association between NAFLD and CAD (Fig. 3b,c). Moreover, given the opposing effects these NAFLD susceptibility genes have on type 2 diabetes risk (Fig. 3d), they also suggest that that plasma lipids have a greater impact on CAD risk than type 2 diabetes. Indeed, we recently expanded our genetic analyses to 12 NAFLD susceptibility genes (identified by either genome-wide association studies for NAFLD or NAFLD-related traits, or meta-analyses) and showed that the effects of these variants on CAD risk are largely accounted for by plasma lipids [55]. We observed a strong relationship between plasma lipids and CAD risk conferred by these NAFLD susceptibility genes [55]. Moreover, since many of these genes, including *PNPLA3* and *TM6SF2*, have also been associated with NASH, it is questionable whether low-grade inflammation plays a major role in the connection between NAFLD and CVD. It should, however, be noted that *PNPLA3* and *TM6SF2* have not been associated with systemic low-grade inflammation [56, 57].

## Clinical implications

The high global prevalence of NAFLD has resulted in an exponential increase in the number and variety of drugs targeting steatosis, NASH and/or fibrosis that have entered Phase II and Phase III clinical trials [58]. Since these agents are aimed primarily at preventing progression to end-stage liver disease and hepatocellular carcinoma, it is important to underscore that the principal cause of death in individuals with NAFLD is CVD [5]. It is therefore essential that any anti-NAFLD drug not only targets NAFLD but also has at least a neutral and preferably a protective effect on CVD events [58]. Given the intertwined relationship between NAFLD and plasma lipid levels (as indicated by the differential effects of NAFLD susceptibility genes on plasma lipids that determine CAD risk [55]), it is strongly recommended that plasma lipid levels are included as an important safety outcome measure in Phase II and Phase III clinical trials.

Another issue of concern is the development of drugs that may have NAFLD as a potential side effect. For instance, glucose-lowering drugs that act on hepatic glucokinase to increase hepatic glucose uptake (e.g. liver-specific glucokinase activators and disruptors of the GKRP–glucokinase complex [59, 60]) could theoretically lead to an increased accumulation of hepatic fat via an increased de novo lipogenesis [61]. By using the common variant in *GCKR* as a model of life-long exposure to a modest increase in hepatic glucokinase activity (Fig. 3a), it can be predicted that it will indeed result in increased de novo lipogenesis and NAFLD, as well as dyslipidaemia and CVD [51, 52]. Of interest, we and others have shown that the effects of this common *GCKR* variant on hepatic fat accumulation and plasma triacylglycerols are more pronounced in conditions of obesity and hyperglycaemia [62, 63]. This would imply that obese individuals and those with poorly controlled type 2 diabetes are more prone to the undesired side effects of liver-specific glucokinase activators. Future studies are warranted to gain more insight into these potential side effects.

## Conclusions and future directions

The MR approach can help to make causal inferences. Although this approach has its specific limitations (e.g. horizontal pleiotropy and statistical power), they can be overcome by combining large datasets using multiple SNPs. The first genetic studies in which NAFLD susceptibility genes were associated with CVD suggest that plasma lipids are an important mediator between both entities; this has important therapeutic consequences. A beneficial effect of a new drug for treating NAFLD may be offset by a greater CVD risk if the drug also increases plasma lipid levels. As hepatic fat accumulation drives the overproduction of lipoproteins, the histological stage ‘simple steatosis’ is not as simple (or benign) as the term suggests. Future studies should be aimed at unravelling the role of other NAFLD-mediated pathways, such as hepatic inflammation, in the pathogenesis of atherosclerosis.

## Electronic supplementary material

Slideset of figures (PPTX 360 kb)

## Abbreviations

CAD: Coronary artery disease
CETP: Cholesteryl ester transfer protein
CRP: C-reactive protein
CVD: Cardiovascular disease
GKRP: Glucokinase regulatory protein
MR: Mendelian randomisation
NAFLD: Non-alcoholic fatty liver disease
NASH: Non-alcoholic steatohepatitis
PAI-1: Plasminogen activator inhibitor type 1
PNPLA3: Patatin-like phospholipase domain-containing protein 3
TM6SF2: Transmembrane 6 superfamily 2
