# Supplementary material for: Non-alcoholic fatty liver disease and cardiovascular disease: assessing the evidence for causality
Source: Diabetologia. 2019 Nov 11;63(2):253–60. doi: 10.1007/s00125-019-05024-3 (PMC6946734; doi:10.1007/s00125-019-05024-3)
Supplement: Supplementary file 1 — (PPTX 360 kb) [file 125_2019_5024_MOESM1_ESM.pptx]

## Slide 1
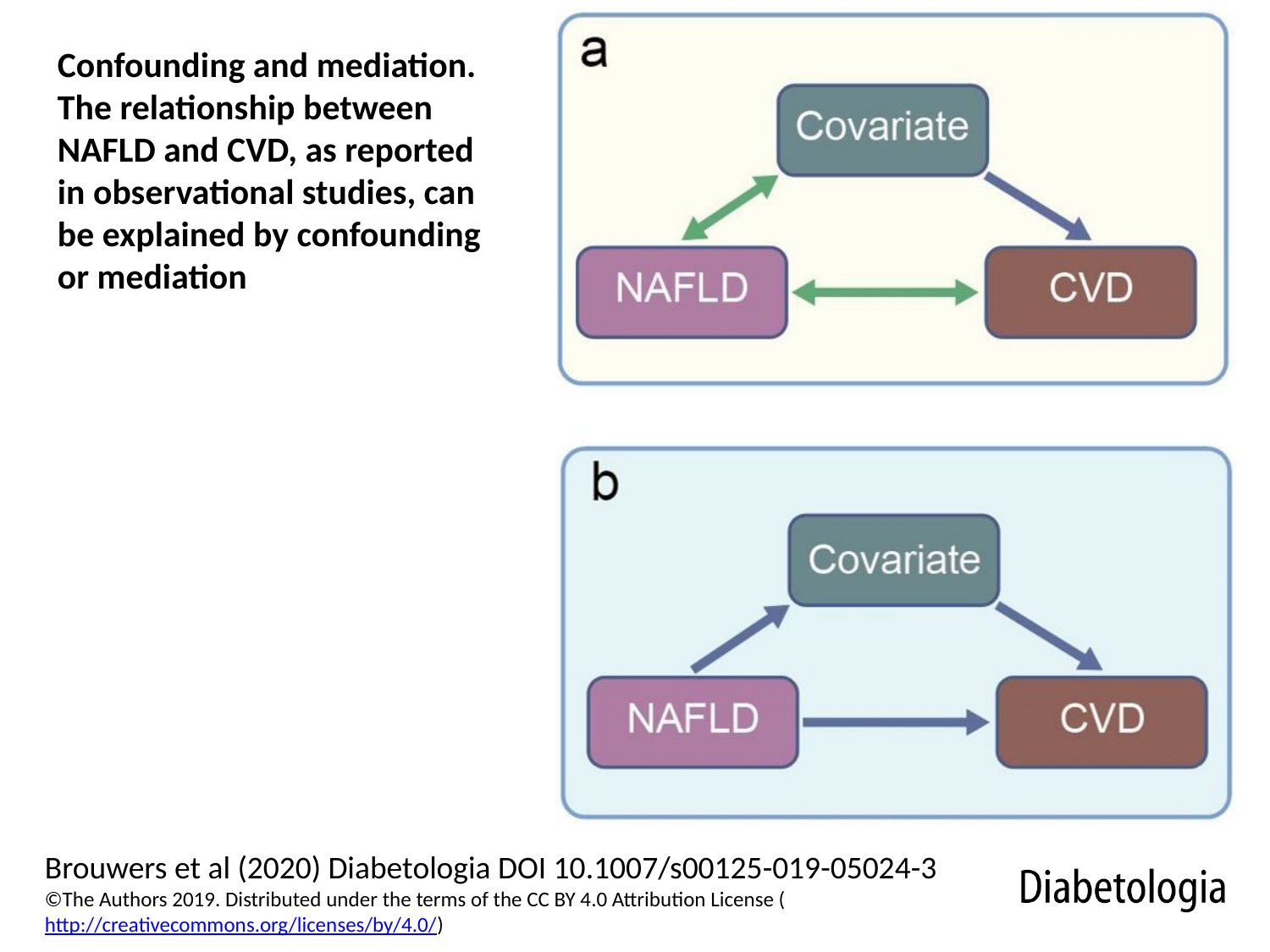

Confounding and mediation. The relationship between NAFLD and CVD, as reported in observational studies, can be explained by confounding or mediation
Brouwers et al (2020) Diabetologia DOI 10.1007/s00125-019-05024-3
©The Authors 2019. Distributed under the terms of the CC BY 4.0 Attribution License (http://creativecommons.org/licenses/by/4.0/)

## Slide 2
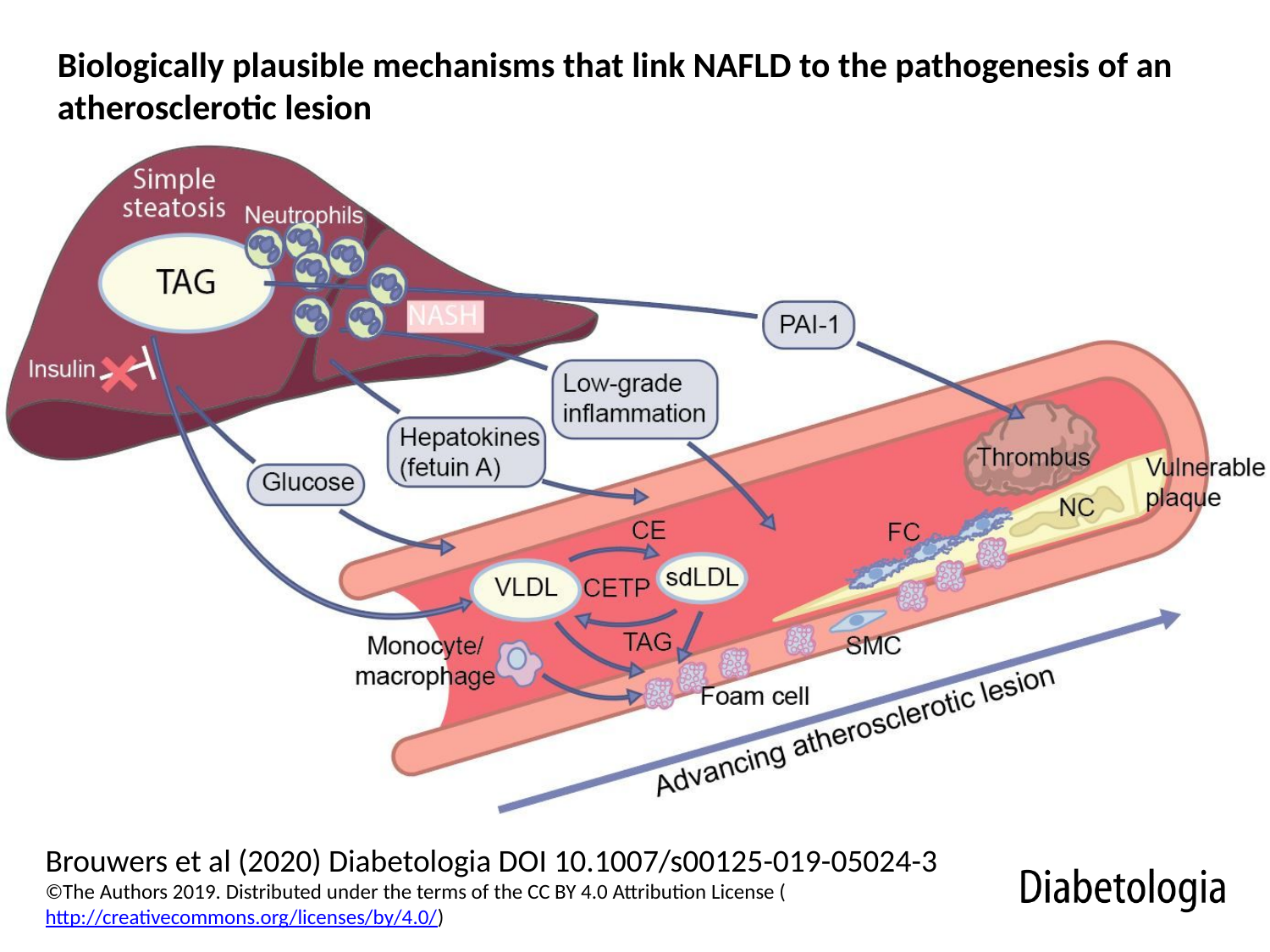

Biologically plausible mechanisms that link NAFLD to the pathogenesis of an atherosclerotic lesion
Brouwers et al (2020) Diabetologia DOI 10.1007/s00125-019-05024-3
©The Authors 2019. Distributed under the terms of the CC BY 4.0 Attribution License (http://creativecommons.org/licenses/by/4.0/)

## Slide 3
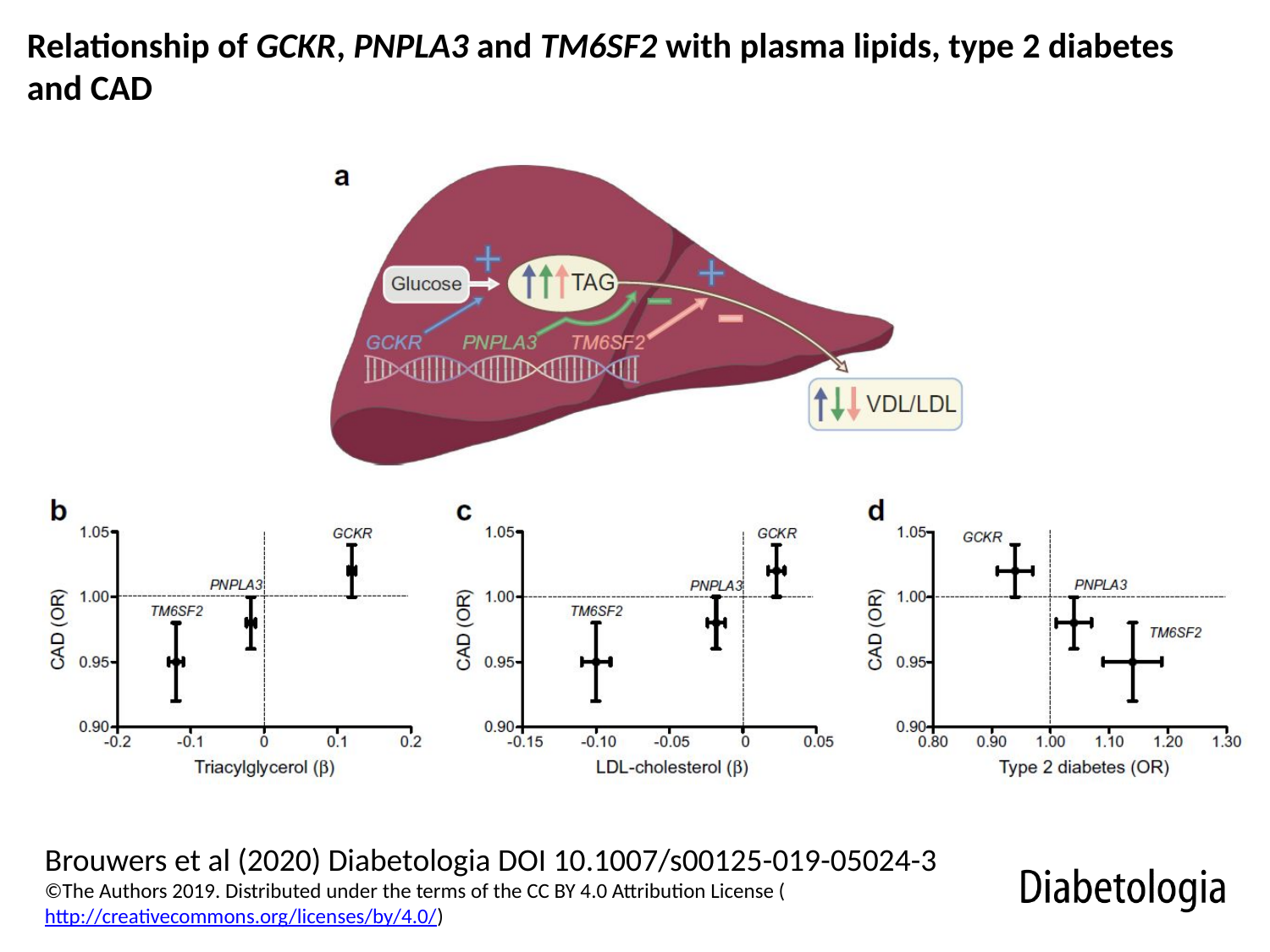

Relationship of GCKR, PNPLA3 and TM6SF2 with plasma lipids, type 2 diabetes and CAD
Brouwers et al (2020) Diabetologia DOI 10.1007/s00125-019-05024-3
©The Authors 2019. Distributed under the terms of the CC BY 4.0 Attribution License (http://creativecommons.org/licenses/by/4.0/)
